# Clinicoanatomic localization of iron-rich gliosis in aphasic presentations of globular glial tauopathy

**DOI:** 10.1093/braincomms/fcag169

**Published:** 2026-06-02

**Authors:** David J Irwin, Sheina Emrani, Daniel T Ohm, Winifred Trotman, Alejandra Bahena, Eric Teunissen-Bermeo, Philip Sabatini, Sandhitsu R Das, Gabor Mizsei, Karthik Prabhakaran, Ranjit Ittyerah, H Branch Coslett, Lauren Massimo, David A Wolk, John A Detre, James C Gee, Edward B Lee, Paul Yushkevich, Corey T McMillan, M Dylan Tisdall

**Affiliations:** Department Neurology, Perelman School of Medicine, University of Pennsylvania, Philadelphia, PA 19104, USA; Department Neurology, Perelman School of Medicine, University of Pennsylvania, Philadelphia, PA 19104, USA; Department Neurology, Perelman School of Medicine, University of Pennsylvania, Philadelphia, PA 19104, USA; Department Neurology, Perelman School of Medicine, University of Pennsylvania, Philadelphia, PA 19104, USA; Department Neurology, Perelman School of Medicine, University of Pennsylvania, Philadelphia, PA 19104, USA; Department Neurology, Perelman School of Medicine, University of Pennsylvania, Philadelphia, PA 19104, USA; Department Neurology, Perelman School of Medicine, University of Pennsylvania, Philadelphia, PA 19104, USA; Department Neurology, Perelman School of Medicine, University of Pennsylvania, Philadelphia, PA 19104, USA; Department Radiology, Perelman School of Medicine, University of Pennsylvania, Philadelphia, PA 19104, USA; Department Neurology, Perelman School of Medicine, University of Pennsylvania, Philadelphia, PA 19104, USA; Department Neurology, Perelman School of Medicine, University of Pennsylvania, Philadelphia, PA 19104, USA; Department Neurology, Perelman School of Medicine, University of Pennsylvania, Philadelphia, PA 19104, USA; Department Neurology, Perelman School of Medicine, University of Pennsylvania, Philadelphia, PA 19104, USA; Department Neurology, Perelman School of Medicine, University of Pennsylvania, Philadelphia, PA 19104, USA; Department Neurology, Perelman School of Medicine, University of Pennsylvania, Philadelphia, PA 19104, USA; Department Radiology, Perelman School of Medicine, University of Pennsylvania, Philadelphia, PA 19104, USA; Department Radiology, Perelman School of Medicine, University of Pennsylvania, Philadelphia, PA 19104, USA; Pathology and Laboratory Medicine, Perelman School of Medicine, University of Pennsylvania, Philadelphia, PA 19104, USA; Department Radiology, Perelman School of Medicine, University of Pennsylvania, Philadelphia, PA 19104, USA; Department Neurology, Perelman School of Medicine, University of Pennsylvania, Philadelphia, PA 19104, USA; Department Radiology, Perelman School of Medicine, University of Pennsylvania, Philadelphia, PA 19104, USA

**Keywords:** globular glial tauopathy, iron, neuroinflammation, MRI, primary progressive aphasia

## Abstract

Globular glial tauopathy is a 4-repeat tauopathy associated with heterogenous clinical syndromes, including primary progressive aphasia. Iron-reactive gliosis in mid-to-deep cortical layers has previously been reported in this disorder, but detailed anatomic localization and its relationship to clinical symptoms is understudied, particularly within the anatomic framework of primary progressive aphasia. In a series of five autopsy-confirmed patients with globular glial tauopathy and one healthy control, we utilize ultra-high-resolution whole-hemisphere *ex vivo* 7 Telsa MRI and digital pathology to study whole-hemisphere and local laminar/cellular patterns of pathology within affected cortex. We find signature laminar patterns of iron-rich gliosis localized to brain regions implicated in distinct clinical aphasia syndromes between patients: patients who presented with non-fluent aphasia had iron-rich gliosis pathology localized to inferior and superior frontal and motor regions while iron-rich pathology was largely localized to the anterior temporal lobe in a patient with the semantic variant. Moreover, in one patient with non-fluent aphasia and additional iron-sensitive 7 Telsa MRI during life, we find evidence of antemortem iron-rich pathology in the same frontal regions observed post-mortem. These data suggest that focal neuroinflammation and iron dysregulation may contribute to the clinical expression of tauopathies and be detectable during life to improve diagnosis.

## Teaser

Irwin and colleagues find anatomic localization of iron-rich gliosis to language-related regions in globular glial tauopathy with primary progressive aphasia using iron-sensitive *ex vivo* 7 Tesla MRI with histological validation and confirmed *in vivo* in one patient who had antemortem 7 Tesla MRI. Neuroinflammation may contribute to clinical symptoms in tauopathies.

## Graphical Abstract

**Graphical Abstract:**
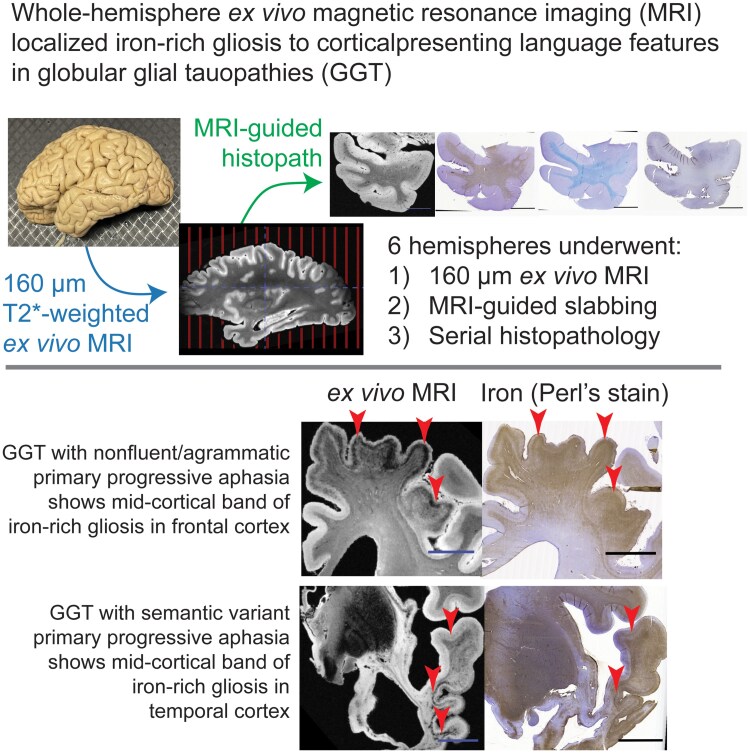

## Introduction

Frontotemporal lobar degeneration (FTLD) is a heterogenous family of neurodegenerative proteinopathies and an important cause of frontotemporal dementia (FTD).^1^ Globular glial tauopathy (GGT) is a form of FTLD characterized by proteinaceous inclusions composed of 4-repeat isoforms of tau predominantly in oligodendrocytes and astrocytes in a globular morphology.^2^ Moreover, additional histopathological findings include microglial activation and iron-retention in a subset of activated microglia and astrocytes,^3^ suggesting neuroinflammation and dysregulation of iron homeostasis may contribute to neurodegeneration.

GGT neuropathology is associated with various FTD clinical presentations, including the behavioural-variant of FTD, primary progressive aphasia (PPA) non-fluent (naPPA) or semantic (svPPA) variants, cognitive/motor syndromes of progressive supranuclear palsy (PSP), corticobasal syndrome and upper-motor neuron predominant form of amyotrophic lateral sclerosis, often with bvFTD.^2-6^ While consensus guidelines include subtyping GGT based partially on the presence of tau pathology within the pyramidal system,^2^ the precise neuroanatomic and cellular contributions to phenotypic heterogeneity of GGT, particularly to diverse features of language impairment, are unclear. Indeed, PPA provides an anatomic framework for testing clinicopathological relationships in FTLD, as each variant anatomically localizes to relatively discrete epicentres of connected regions in the language network, with most prominent disease in the left anterior temporal cortices in svPPA^7^ compared with greatest pathology in the left inferior and dorsolateral frontal cortices in naPPA.^8^

Using ultra-high-resolution *ex vivo* 7T magnetic resonance imaging (MRI), we developed a pipeline to routinely acquire whole-hemisphere imaging of post-mortem FTLD human brain tissue enabling us to visualize the regional distribution of pathology at the mesoscopic scale.^9^ Our MRI protocol focuses on T_2_*-weighted (T_2_*w) imaging, a contrast that has been shown in post-mortem brain tissue to be driven largely by iron content. In healthy brain tissue the primary source of iron-driven contrast arises in myelin from oligodendrocytes, leading to T_2_*w contrast visualizing healthy cortical myeloarchitecture.^10^ Our group and others observe additional T_2_*w hypointensity from pathological iron accumulation in reactive glia in FTLD, where GGT and other sporadic tauopathies have irregular mid-to-deep layer hypointense cortical bands that are largely distinct from laminar patterns observed in FTLD-TDP, AD and healthy adult brains,^9,11,12^ but also recently reported in familial forms of FTLD.^13^

Here, we study histopathology, along with *ex vivo* and antemortem MRI, in a series of well-characterized patients with GGT who presented with PPA to test the hypothesis that this iron-rich gliosis is predominantly localized to regions contributing to presenting clinical features of PPA. We find converging radiological and neuropathological evidence to suggest that cellular patterns of neuroinflammation and iron dysregulation post-mortem mark clinically-relevant regions in GGT, and in one patient with available antemortem iron-sensitive 7T MRI imaging, we detect a similar neuroanatomic pattern of pathological iron during life.

## Materials and methods

Patients selected had a primary neuropathologic diagnosis of GGT with *ex vivo* 7T MRI-guided histology enrolled in observational research at the Penn Frontotemporal Degeneration Centre (*n* = 5). We include one additional healthy control hemisphere with limited age-related pathology. Clinical diagnoses were established using clinical criteria.^14-16^ Neuropathologic diagnoses were performed using established methods and neuropathologic diagnostic criteria.^2,17^ Clinical and diagnostic neuropathologic data was obtained from the Penn Integrated Neurodegenerative Disease database.^18^ Ordinal ratings for morphologic features of GGT^2^ were performed on diagnostic slides using a general ordinal rating scale (i.e. 0 = none/rare, + = mild, ++ = moderate, +++ = severe) per criteria^17^ (Table 1, Supplementary Figs 1–2).

**Table 1:** Patient clinical and pathological data

Clinical diagnosis	Patient #1 naPPA/CBS	Patient #2 naPPA/CBS	Patient #3 naPPA/PSP	Patient #4 naPPA/PSP	Patient #5 svPPA/bvFTD	Patient #6 Control
**Sex**	Male	Female	Male	Female	Male	Female
**Age at Onset (y)**	72	68	68	58	61	-
**Age at Death (y)**	76	74	74	65	70	67
**Disease Duration (y)**	5	6	7	7	9	-
**Brain Weight (g)**	1316	1179	1180	1080	1208	1060
**PMI (hours)**	18	19	18	30	16	19
**Fixation time before scan(days)**	185	97	64	80	283	78
**ADNC Stage**	Low	Low	None	Low	Low	None
**Co-Pathology**	None	None	None	None	CAA	None
**GGT subtype**	II	II	II	III	III	None
**Oligo. tau**	+++	+++	+++	++	+	None
**Astrocyte tau**	++	+	+	+++	+++	None
**Anterior horn tau**	+	+	+	NA	NA	NA
**CST tau**	++	++	+++	+++	++	None
**Hemisphere**	L	L	L	L	L	R
	**Onset of Clinical Features** ^a^					
**Imp. Grammatical Expression/Comprehension**	Onset	2	Onset	Onset	-	-
**Non-Fluent Speech**	Onset	Onset	Onset	Onset	-	-
**AoS**	2	3	5	4	-	-
**Imp. Repetition**	-	3	5	3	-	-
**Imp. Single Word** **Comprehension**	-	-	-	-	Onset	-
**Surface Dyslexia**	-	-	-	-	2	-
**Behavioural Disinhibition**	4	4	Onset	5	5	-
**Apathy/Inertia**	4	4	5	5	8	-
**Loss of Empathy**	-	-	4	-	8	-
**Compulsive Rituals**	-	-	-	Onset	Onset	-
**Hyperorality**	4	-	Onset	5	8	-
**Parkinsonism**	2	3	4	3	-	-
**Lateralized Apraxia**	-	-	4	3	-	-
**Supranuclear Gaze Palsy**	3	2	-	3	-	-

^a^Each cell of clinical symptoms denotes the years from the time of onset for the emergence of clinical feature in the clinical record, Onset = a presenting clinical feature of disease; − = clinical feature not present in the medical record.

Disease Duration = year of death-year of reported onset, CST = corticospinal tract, PMI = post-mortem interval, y = years, ADNC = Alzheimer’s disease neuropathologic change, CAA = Cerebral amyloid angiopathy, Imp = Impaired, AoS = Apraxia of Speech, NA = not available.

Whole-hemisphere T_2_*w *ex vivo* 7T MRI imaging was performed at 160 µm isotropic resolution prior to histologic processing of samples using previously reported methods^9,19^ (Patient #2 the temporal lobe was removed prior to scanning; Patient #1MRI was obtained at 280 µm resolution). Full details of the multi-echo gradient-recalled echo sequence for each subject are given in Supplementary Table 1, but in all cases the echo closest to 20 ms provided a T_2_*w image that was used for subsequent visual inspection and analysis.

Please see^19^ for an overview of our MRI-guided histopathologic sampling. Based on visual inspection of T2*w *ex vivo* MRI, we focused on a block containing superior prefrontal [BA8] and adjacent primary motor cortex [BA4] and a block containing anterior inferior temporal cortex [BA20] in paraffin-embedded tissue using our previously described MRI-guided sampling.^11^ Paraffin tissue blocks (50 × 75 mm) were sectioned at 20 µm thickness and immunostained for phosphorylated-tau (S202/T205; AT8, 1:500, Thermo Fisher)^20^ and chemically stained for iron using Perl’s stain with DAB enhancement^21,22^ and Luxol-fast blue for myelin with cresyl violet counterstain as described.^9,11^ Whole slide images of histology were obtained from a Huron Tissue Scope LE120 (Huron Digital Pathology, St. Jacobs ON) at 20×.

To confirm cellular contributions to iron-reactivity, we dissected a subregion of BA4 and BA20 from a subset of semi-adjacent 50 × 75 mm sections above, cut at 10 µm thickness onto traditional 25 × 75 mm slides for multi-label immunofluorescence (IF) experiments (see Supplementary Methods).

Antemortem T_1_-weighted (T_1_w) and T2-weighted (T_2_w) MRI data was acquired for patients 1–4 on a 3T whole-body MRI scanner (MAGNETOM Prisma, Siemens Healthineers). T_1_w images were acquired using 3D MPRAGE sequences while T_2_w images were acquired using either axial 2D FLAIR or Turbo Spin Echo (TSE) pulse sequences. Patient #4 additionally had T_2_*w imaging acquired on a 7T whole body MRI scanner (MAGNETOM Terra, Siemens Healthineers). Variation in acquisition parameters between patients was a result of their participation in various generations of our research imaging protocol; all parameters are summarized in Supplementary Table 2.

### Statistical analysis

Please see supplementary methods for details on figure generation and laminar quantification. Signal intensity between MRI and DAB in corresponding histology for each sample were correlated by calculating the Pearson correlation coefficient (R) between binned data-points (100 per image) in normalized space from pial surface to grey-white junction for each modality with statistical threshold of *P* = 0.05.

A limited set of data was previously reported (patients #1–2).^9^ All procedures were performed with informed consent in accordance with the Penn IRB.

## Results

Clinical and neuropathologic data are included in Table 1 and representative photomicrographs in Supplementary Figs 1–2. Patients #1–4 presented with an agrammatic non-fluent speech disorder consistent with naPPA, while patient #5 had fluent speech with single-word and object semantic knowledge loss consistent with svPPA. Associated motor features of lateralized apraxia and Parkinsonism consistent with CBS^16^ later emerged in naPPA patients #1–2 while patients #3–4 developed additional occulomotility and motor difficulties consistent with PSP.^15^ Patient #5 did not develop motor symptoms. Neuropathological examination in patients #1–3 found prominent globular tau reactivity in oligodendrocytes, and to a lesser extent in astrocytes, in a widespread distribution which included motor cortex, corticospinal tract (CST) and the anterior horn of the spinal cord, consistent with GGT type II. Patient #4 had a similar distribution but more predominant globular astrocyte burden, consistent with GGT type III. Patient #5 tau pathology was widespread, including the CST, with highest burden in temporal cortex grey matter, largely in the form of globular astrocytes, most consistent with type III^2^ (Supplementary Table 3, Supplementary Figs 1–2).

**Figure 1:**
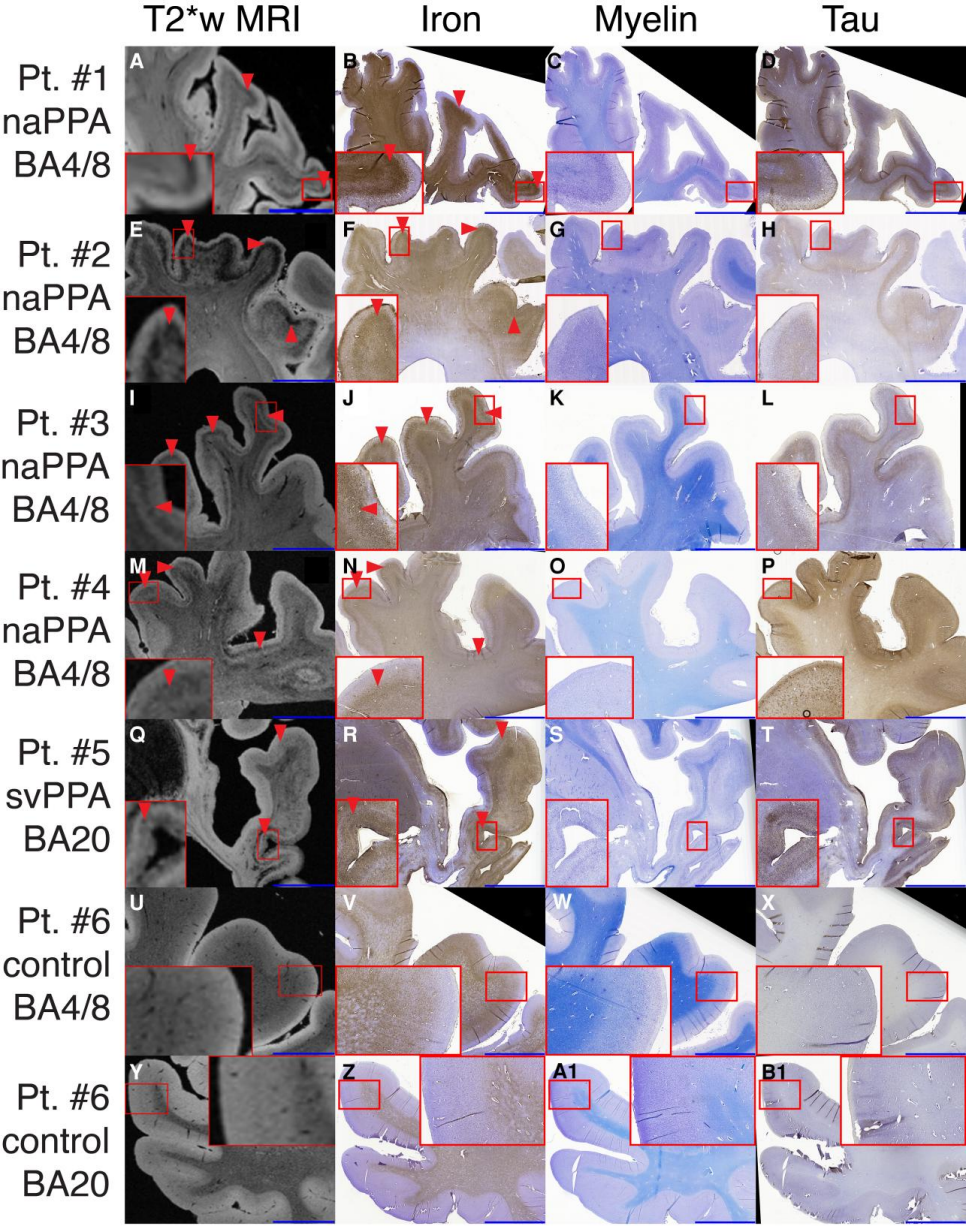
*Ex vivo* 7T T_2_*w MRI and corresponding tissue histopathology. Histopathological correspondence of T_2_*w MRI hypointense banding in naPPA patients (**A, E, I, M**) in BA 4/8 and svPPA patient (**Q**) in BA20 compared with control hemisphere BA4/8 (**U**) and BA 20 (**Y**). Serial sections corresponding to 7T MRI stained with modified Perl’s stain with DAB for iron (**B, F, J, N, R, V, Z**), luxol-fast blue for myelin (**C, G, K, O, S, W, A1**) and phosphorylated tau (**D, H, L, P, T, X, B1**). Hypointense MRI signal in GGT brains (arrows in **A, E, I, M, Q**) corresponds to iron-rich glia (arrows in **B, F, J, N, R**) accompanied by degeneration of intracortical myelin, neurodegeneration and tau pathology not observed in control tissue. Samples are oriented in radiological convention for left/right. Scale bar (blue) = 1 cm. Red box inset depicts high-magnification (3×) of laminar profile in representative cortex for MRI and histology.

Whole-hemisphere *ex vivo* T_2_*w MRI in GGT patients compared with control tissue revealed laminar microstructural changes characterized by the variably obscured normal regional myeloarchitectural laminar patterns that were largely replaced by irregular hypointense banding in mid-to-deeper cortical layers with extension into adjacent and deep white matter (WM). This pattern also included hypointense signal surrounding larger vessels in WM superimposed on relative overall WM hyperintensity (WMH) in diseased regions (Fig. 1, Supplementary Fig. 3). Patients #1–4 with naPPA showed this irregular laminar hypointensity in anterior superior frontal gyrus (BA 8) extending into primary motor (BA4), middle frontal gyrus and the ascending ramus of pars opercularis of Broca’s area. Patients #3 and 4 had additional extension into pars triangularis of Broca’s area. The most intense banding pattern was observed in the superior and middle aspects of the primary motor homunculus. Patient #5 with svPPA showed a similar laminar pattern of irregular hypointense banding, but in contrast, this pathology was most intense in the anterior temporal lobe, including the ventral and middle temporal gyri (BA20), along with severe localized WMH with superimposed hypointensity (Fig. 1). In severely degenerated areas, there was also a variable additional upper cortical layer hypointense band. There was extension of less prominent hypointense mid-layer banding/speckling both superiorly into the ventral orbitofrontal and insular cortices with subtle abnormalities in the inferior and middle frontal gyri and posteriorly into the temporal-parietal cortex (Supplementary Fig. 3).

Corresponding histopathological examination of BA4/8 and BA20 showed differential findings across the patients, consistent with antemortem symptoms and *ex vivo* T_2_*w imaging above. BA4/8 in naPPA patients #1–4 demonstrated variable abolishment of normal cortical myeloarchitecture on myelin stain and vacuolization of mid-to-deep cortical layers with a high burden of tau pathology. This was accompanied by pathologic iron-reactivity not observed in control tissue, largely in the form of activated glia in mid-to-deeper cortical layers (III-VI), often in proximity to vessels in deep GM and in associated WM (Fig. 1). Available BA20 in naPPA showed low tau pathology and normal myeloarchitecture corresponding to iron-reactivity largely restricted to healthy oligodendrocytes and associated intracortical myelin, similar to control tissue (Supplementary Fig. 4). In contrast, patient #5 with svPPA showed highest GGT tauopathy in BA20 with severe degeneration and iron-rich glia variably in upper layers and largely in deeper layers and juxtacortical U-fibers (Fig. 1), whereas in BA4/8 tau pathology was low and iron-reactivity reflected in healthy myeloarchitecture as in our control tissue (Supplementary Fig. 4).

Finally, we performed a quantitative analysis of the laminar distributions of 7T T2*w MRI intensity and corresponding DAB-enhanced iron-stained tissue in the regions of interest above. This analysis found good correlation of both modalities along the cortical depth (average Pearson *R* = −0.90 ± 0.08, *n* = 7 image-pairs, *P* < 0.001 with draw-re-draw validation −0.78 ± 0.16 *n* = 7 image-pairs, *P* < 0.001; Supplementary Fig. 5), confirming our qualitative observations.

**Figure 2:**
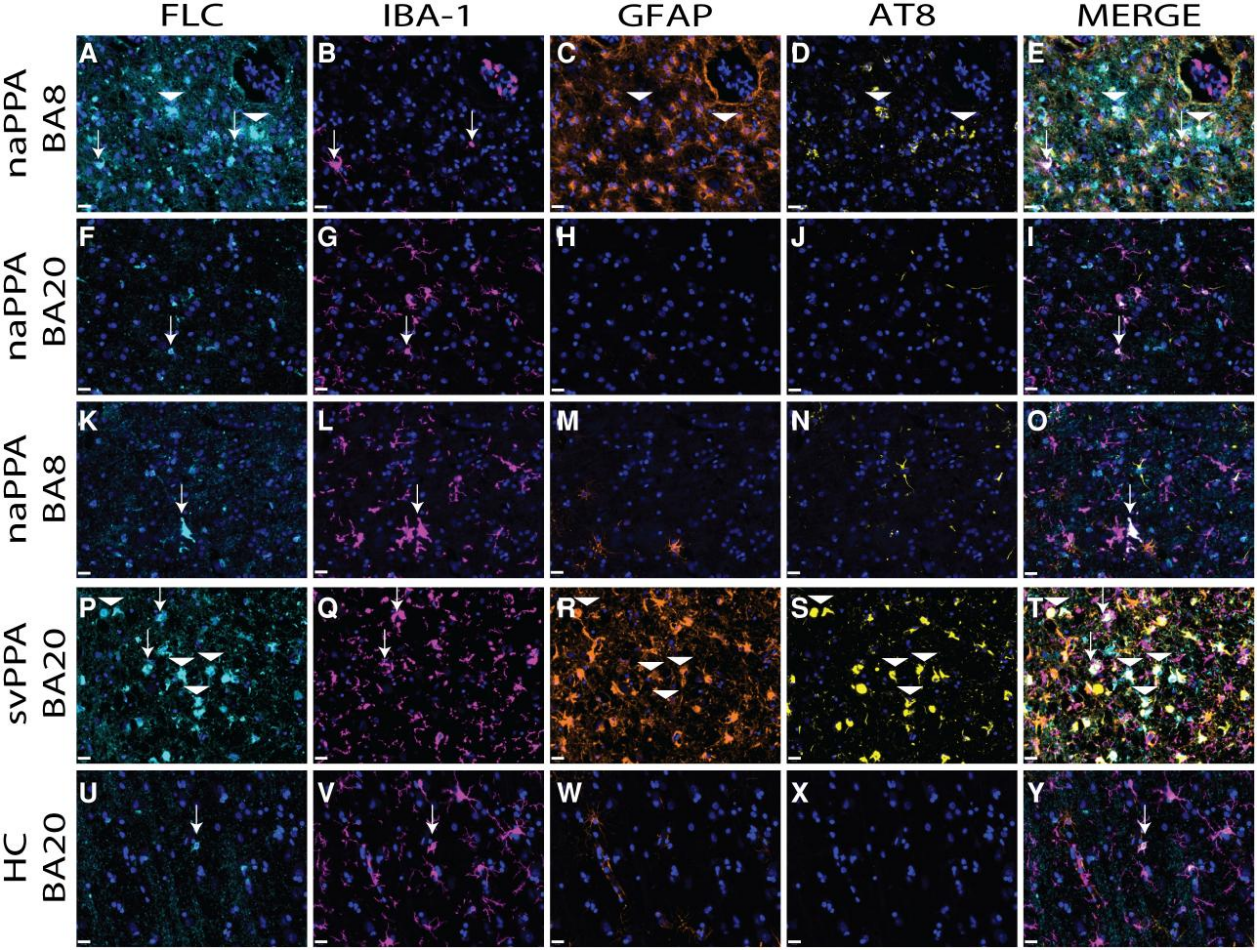
**Cellular co-localization of iron in GGT.** Photomicrographs depict representative images of areas of high iron-rich gliosis in GGT patient #3 with naPPA in BA8 (**A-E**) and GGT patient #5 with svPPA in BA20 (**P-T**), as well as the low pathology region BA20 in GGT patient #3 with naPPA (**F-I**), low pathology BA8 region in GGT patient #5 with svPPA (**K-O**) and healthy control (HC) patient #6 in BA20 **(U-Y)** immunostained for ferritin light chain (FLC; **A, F, K, P, U**), microglia (ionized calcium-binding adaptor molecule 1, IBA-1; **B, G, L, Q, V**), reactive astrocytes (glial fibrillary acidic protein, GFAP; **C, H, M, R, W**) and phosphorylated tau (AT8, **D, J, N, S, X**; merge all channels **E, I, O, T, Y**). Co-localization experiments find high levels of iron-reactivity in clinically-relevant regions in GGT cases with PPA labelled by FLC co-localize with IBA-1 reactive microglia (arrows) and variably GFAP-reactive astrocytes often with tau pathology (arrowheads). Low tau pathology regions (naPPA BA20, svPPA BA8) showed scant FLC-reactive microglia similar to control tissue. Scale bars = 20 μm.

Multi-label IF experiments confirmed the colocalization of iron reactivity using FLC immunostaining which co-labelled a subset of IBA-1-reactive microglia, as well as tau-positive astrocytes with variable GFAP-reactivity with highest abundance in mid-to-deep layers (Fig. 2).

**Figure 3:**
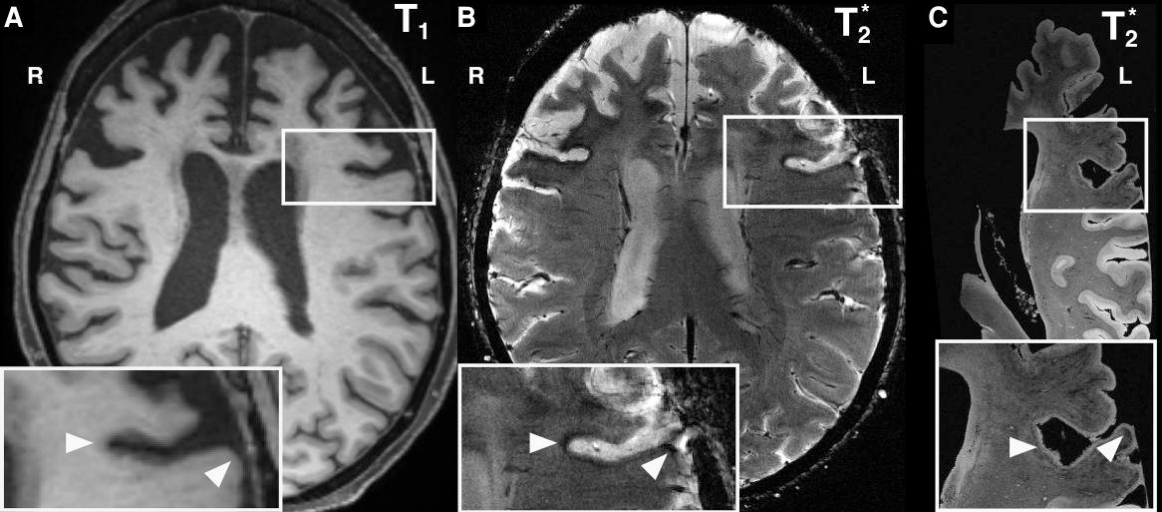
Correspondence of antemortem 7T T_2_*w MRI hypointensity with iron-rich gliosis on post-mortem ex vivo 7T T_2_*w MRI. (**A**) Patient #4 with naPPA antemortem three Tesla T1-weighted (3T_1_w) MRI, (**B**) antemortem 7T T_2_*w MRI and (**C**) post-mortem *ex vivo* 7T2*w MRI axial views and zoomed regions (insets). Note the correspondence of hypointense cortical signal in BA8 on *in vivo* 7T T_2_*wMRI (**B** arrows) to iron-rich hypointense banding on corresponding *ex vivo* 7T2*w MRI (**C** arrows), while 3T_1_w MRI does not show hypointensity and instead shows reduced GM/WM contrast (**A** arrows).

Antemortem structural MRI (available in patients #1–4) demonstrated variable frontal cortical atrophy and WMH (Supplementary Fig. 6) that were largely left-lateralized. Patient #1 had extensive WMH on T_2_w corresponding to hypointense regions on the T_1_w scan, while patients #2–4 had minimal/no WMH or other white matter abnormalities on T_2_w or T_1_w imaging (Supplementary Fig. 6). Patients #1–4 had variable thinning of the anterior to middle segments of the corpus callosum. An iron-sensitive 7 T T_2_*w sequence obtained during life in patient #4 demonstrates strong overlap between regions of iron-rich pathology in both antemortem and *ex vivo* MRI (Fig. 3).

## Discussion

Our novel approach, integrating microscopic pathology in the context of gross whole-hemisphere imaging, reveals important new data about the spatial distribution of pathology and the cellular contributions to clinical heterogeneity of GGT. In each patient, *ex vivo* T_2_*w MRI showed a signature pattern of frank hypointensity largely in middle-to-deep cortical layers and adjacent WM. This pattern in turn, corresponded to iron-rich gliosis on histology, and to a similar anatomic distribution of hypointensity visualized antemortem using iron-sensitive T_2_*w 7T MRI in patient #4 with available data. Our unique radio-pathological study of rare GGT cases suggests that MRI metrics of iron-retention and laminar disease developed with sufficient resolution *in vivo* could provide diagnostically relevant information not captured by traditional MRI-based macrostructural measurements.

While each GGT patient had an overall similar laminar profile of deep-layer iron-rich pathology, the regional distribution varied according to the presenting clinical features of naPPA compared with svPPA (Figs 1 and 3). Interestingly, temporal cortices with severe degeneration in svPPA patient #5 had a variable additional upper layer band, which may be related to stage of disease or region-specific factors. Critically, in all five patients the regional distribution of this stereotypic iron-rich gliosis was closely linked to regions with severe neurodegeneration, in contrast to the observed more widespread distribution of tau pathology of varying severity (Supplementary Table 3). Thus, incorporation of histological measures of gliosis and iron-laden neuroinflammation may enhance future clinicopathological correlation and histopathological staging for GGT. Indeed, one study found antemortem hypointensity on iron-sensitive MRI corresponding to iron-reactive glia on histology in the motor strip in a GGT patient who presented with upper-motor neuron signs^3^ and a recent post-mortem study of iron reactivity and gene expression in progressive supranuclear palsy (another 4-repeat tauopathy) found retention of iron predominantly in tau-positive astrocytes near large vessels in early-affected subcortical regions.^23^ Together with our observations here, these data suggest that neuroinflammation, iron dysregulation and possible blood-brain barrier disruption may occur in regions affected early in the neurodegenerative process to contribute to the presenting clinical symptomatology in GGT and related tauopathies.

Interestingly, GGT patients with naPPA had greater relative iron-reactivity in dorsolateral frontal and motor regions compared with Broca’s area (Fig. 1, Supplementary Fig. 3). Indeed, naPPA atrophy includes a distributed network of connected left-hemispheric peri-Sylvian regions^8^ that also include the superior dorsolateral frontal regions studied here, which contribute to grammatical expression.^8,24^ We previously found partially distinct regional and laminar patterns of iron-rich gliosis in GGT compared with other clinical/pathological subtypes of FTLD.^9^ Thus, future work using this approach can compare mesoscopic patterns of GGT cellular pathology to other forms of FTLD within each clinical PPA syndrome to test for GGT-specific neuronal vulnerabilities within the language network, such as the predominant dorsal frontal and motor involvement observed here in naPPA-GGT.

Notably, these laminar patterns of iron-rich gliosis were not evident on available structural T_1_w or T_2_w antemortem scans (Supplementary Fig. 6). Autopsy-confirmed *in vivo* imaging in GGT is extremely rare, but existing structural MRI studies largely focus on T_1_w and/or T_2_w sequences and generally find correspondence of cortical atrophy to clinical features.^25^ There are mixed reports of WMH in GGT^25-28^ and antemortem MRI data in our patients was similarly mixed, with stereotypical findings of intensity changes in peripheral WM^27^ in only one of four patients with available data, while corpus callosum thinning was present in all four patients. In one report WMH emerged late in disease,^28^ thus there may be complex dynamic features of neurodegeneration, along with heterogeneity of GGT subtypes, that contribute to variable presence of WMH in GGT.

Importantly, our most recent autopsy patient #4 had an antemortem T2*w 7 T MRI sequence acquired. This *in vivo* imaging shows a similar anatomic pattern of pathologic hypointensity providing proof-of-concept for the ability to detect and track this histopathologic feature of GGT during life (Fig. 3). Thus, iron-sensitive MRI imaging at sufficient laminar resolution can potentially provide complementary data reflecting cellular pathology in GGT to traditional MRI volumetrics, which thus far have not shown diagnostic ability to differentiate GGT from other forms of FTLD.^25^ Indeed, laminar-scale (e.g. 250 μm) resolution has been recently demonstrated with *in vivo* MRI using focal line-scan techniques, which could be achieved in clinically feasible scan-times.^29,30^

While our multi-modality approach facilitated a rigorous evaluation of heterogenous clinical presentations of GGT, it is an extremely rare disorder, and we lacked patients with other syndromes including ALS-FTD and patients with GGT type I. Future work in a larger dataset to analyze both iron-sensitive *in vivo* and *ex vivo* MRI with histopathological validation, can further elucidate progression of iron-rich gliosis in GGT and related tauopathies. These limitations notwithstanding, our data suggest neuroinflammation and iron dysregulation may play an important role in the neurodegenerative process of vulnerable brain regions to contribute to clinical heterogeneity of GGT; potential detection of these processes *in vivo* could inform a molecular diagnosis in FTD spectrum disorders.

## Supplementary Material

fcag169_Supplementary_Data

## Data Availability

All MRI imaging data that support this study are openly available via Dryad at the following link: https://doi.org/10.5061/dryad.dbrv15fgk. All histopathology data that supports this study and tissue are available upon reasonable request from the authors, conditional on establishing a formal data sharing agreement with the University of Pennsylvania. Code for figure generation and laminar analysis and corresponding images are available at GithHub at the following link: https://github.com/PICSL-FTDC-Computational-Pathology/FTDC_GGT_paper/tree/main.
