# Supplementary material for: Clinicoanatomic localization of iron-rich gliosis in aphasic presentations of globular glial tauopathy
Source: Brain Commun. 2026 Jun 2;8(3):fcag169. doi: 10.1093/braincomms/fcag169 (PMC13227947; doi:10.1093/braincomms/fcag169)
Supplement: fcag169_Supplementary_Data [file fcag169_supplementary_data.pdf]

Supplementary Materials

Clinicoanatomic localization of iron-rich gliosis in aphasic presentations of globular glial tauopathy

David J. Irwin[1*], Sheina Emrani[1], Daniel T. Ohm[1], Winifred Trotman[1], Alejandra Bahena[1], Eric Teunissen-Bermeo[1], Philip Sabatini[1], Sandhitsu R. Das[1], Gabor Mizsei[2], Karthik Prabhakaran[1], Ranjit Ittyerah[1], H. Branch Coslett[1], Lauren Massimo[1], David A. Wolk[1], John A. Detre[1,2], James Gee[2], Edward B. Lee[3], Paul Yushkevich[2], Corey T. McMillan[1], M. Dylan Tisdall[2*]

[1]Neurology, Perelman School of Medicine, University of Pennsylvania

[2]Radiology, Perelman School of Medicine, University of Pennsylvania

[3]Pathology and Laboratory Medicine, Perelman School of Medicine, University of Pennsylvania

Supplement: Methods=1, Tables=3 , Figures=6

Supplementary Methods.

MRI-Histology Registration

To align histopathology and corresponding ex vivo MRI to generate Figure 1, we first used the interactive tool within the image registration function in ITK-SNAP[1] (version 4.2.0) to reorient and align tissue from whole slide images of serial sections stained for iron (Modified DAB-enhanced Perl's stain), myelin (luxol fast-blue) and phosphorylated tau (AT8) for each case. Each slide was rotated and scaled to match the tau antibody (AT8) slide, and subsequently interpolated to apply the same resolution to the reference image (Tau slide). The corresponding *ex vivo* MRI image was also captured using ITK-SNAP through reorienting the *ex vivo* MRI to match the cutting plane of the block taken from histology; the MRI image plane was then manually aligned to the histology slide by scrolling along the anterior-posterior axis in the coronal view using the same interactive tool within the image registration. Slides and corresponding MRI images were then exported from ITK-SNAP using the export function as PNGs and imported into Photoshop (version 25.12.2) to rescale the PNG images to have consistent resolutions for all MRI and histology slides and crop images to minimize non-tissue background. Scale bars were added in photoshop based on the fixed resolution of the underlying histology and MRI images from paired exported images with scale bars embedded in the image. Custom scripts available on GitHub (see data availability statement) exported images and high magnification images (3x zoom from main figure) that were captured from WSI in representative gyri for inset images to demonstrate laminar profiles of each stain and from corresponding ex vivo MRI image. These PNGs were imported to Adobe Illustrator (version 28.7.7) for figure composition and panel labels.

*Laminar Profiles of Ex vivo T2*w MRI and corresponding Iron-stain histology*

Cortical regions of interest (ROIs) were manually generated from rescaled PNG whole-slide images (WSI) of histology sections stained for DAB-enhanced iron and in corresponding postmortem 7T T2*w MRI PNG files. ROIs are defined in ITK-SNAP[1] (version 4.2.0) by 4 annotations: 2 lines perpendicular to the cortex surface which define the start and end of the ROI; 2 lines normal to the cortical surface are the boundaries of the upper pial surface and the deeper grey/white matter boundaries. Each of these boundaries intersect to create a 4 sided ROI for analysis. Manual annotations were performed by two separate investigators for draw-re-draw validation.
Each DAB-enhanced iron image undergoes color deconvolution so that both images MRI and histology images are grayscale. The intensity of the color deconvoluted histology grayscale image of DAB is the inverse relative to the expected MRI intensities (i.e. hypointense MRI corresponds to short T2* expected in regions of high iron, while hyperintense DAB labelling corresponds to increased iron).

We then resample both images using the annotations to a normalized cortical space. This is achieved via a sampling grid derived from the 4 annotations drawn by the user. Each column in the sample grid corresponds to a path of coordinates in the original image to travel through to get from the upper pial surface to deep grey/white matter boundary. Each path is calculated by a weighted average of the parameters of the starting and ending annotations, clipped by upper pial surface and the deeper grey/white matter boundaries. (Example image shown below, and equations which define the sample grid). This method generalizes to higher dimensions (i.e. curved annotations) however for simplicity of explanation and analysis

we used linear equations for our annotations.

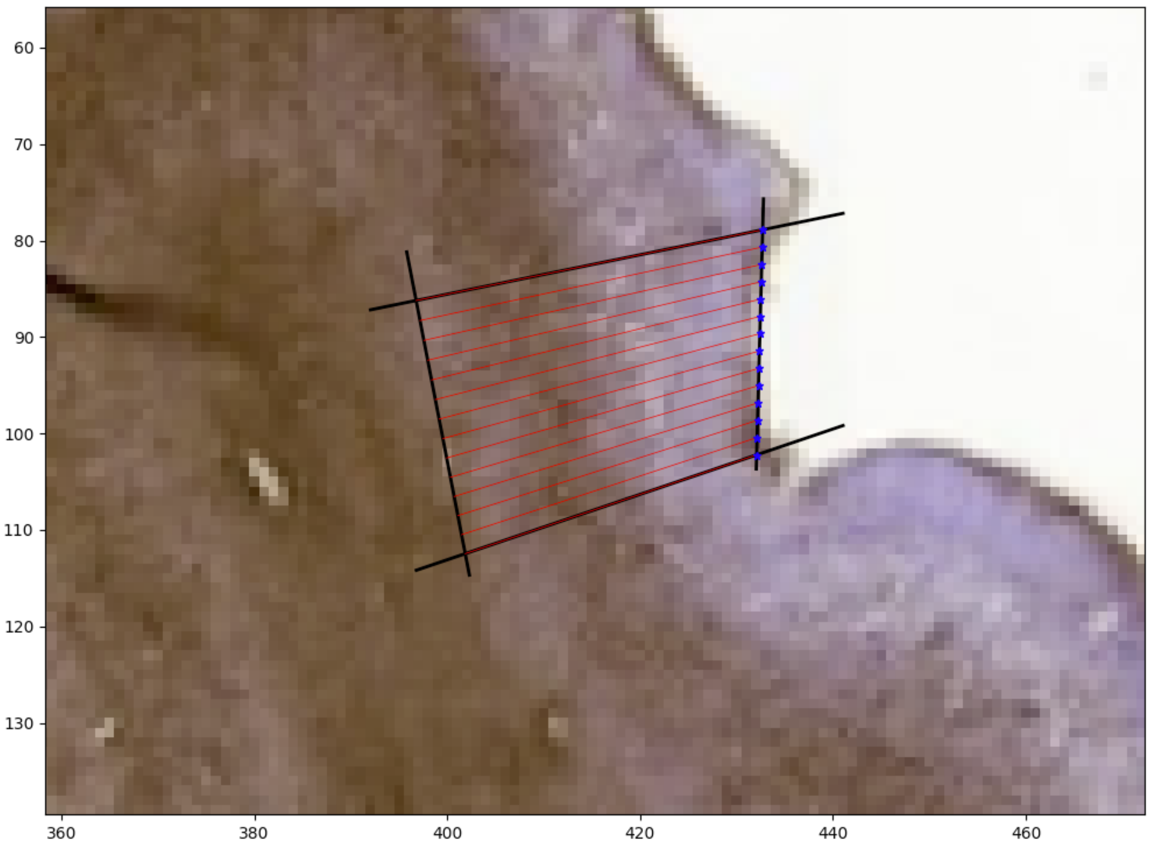

$$y_{csf} = m_{csf} * x + b_{csf}$$
$$y_{wm} = m_{wm} * x + b_{wm}$$
$$x_{start} = m_{start} * y + b_{start}$$
$$x_{end} = m_{end} * y + b_{end}$$

$$G(x, y); 0 \leq x \leq 1; 0 \leq y \leq 1$$
$$G(x, 0) = y_{csf}$$
$$G(x, 1) = y_{wm}$$
$$G(0, y) = x_{start}$$
$$G(1, y) = x_{end}$$
$$G(x, y) = [x * x_{start} + (1 - x) * x_{end},\ \ y * y_{csf} + (1 - y) * y_{wm}]$$

Once we have both modalities in the same normalized cortical space, we take the median value at 100 binned points across the cortex to derive an intensity curve based solely on cortical depth. These curves were then correlated by

calculating the Pearson correlation coefficient (R) for overall signal intensity (Negative correlation is expected due to the inverse intensity relationship between DAB-iron stain and MRI).

Multiplexed Immunoflouresence

To confirm cellular contributions to iron-reactivity, we dissected a subregion of BA4 and BA20 from a subset of semi-adjacent 50x75mm sections above, cut at 10μm thickness onto traditional 25x75 mm slides for multi-label immunofluorescence (IF) experiments. We used primary antibodies specific for Ferritin Light Chain (FLC 1:400; Abcam, #ab21866) , glial fibrillary acidic protein (GFAP 1:50; GeneTex, #GTX636725) IBA-1 (1:50; GeneTex, #GTX635363) and AT8 (S202/T205; clone AT8, Thermo Fisher 1:100) conjugated to complementary oligonucleotide-bound fluorophores (FLC=647; GFAP, IBA-1= 750; AT8=550; Akoya Biosciences, Marlborough, MA). Slides were hydrated, antigen retrieval performed with 20 minutes of pressure-cooker at 115C degrees, followed by incubation overnight with a cocktail of primary antibodies in blocking buffer overnight at 4C. Next, fixation was performed with 1.6% paraformaldehyde in blocking buffer for 10 min at room temperature, slides incubated in methanol at 4C for 5 min to quench autofluorescence, followed by fusion reaction performed with reporter fluorophores and imaged at 20x using an Akoya Phenocycler platform (Akoya Biosciences). Exposure times included 75msec for FLC and 150msec for GFAP and AT8, 300ms for IBA-1.

Figure 2 photomicrographs of multiplexed immunofluorescence images were obtained using QuPath version 0.50[2]. Whole slide images were reviewed, and optimum thresholds were defined empirically to visualize each stain applied across images using the "brightness & contrast function" and visualization of stain intensity histogram in log form. Settings included: DAPI channel minimum= 457.1, channel maximum= 6592.5, viewer gamma=1; IBA-1 channel Minimum= 5.31, channel maximum=1173, viewer gamma=1; AT8 channel minimum= 21.6, channel maximum=7170, viewer gamma=1; GFAP channel minimum= 326.5, channel maximum=4020.1, viewer gamma=1; Ferritin channel minimum= 197, channel maximum=1858.4, viewer gamma=1. Representative images were captured at 40x using the "export snapshot function"… "current viewer content" in PNG format. PNG images were imported to Adobe Illustrator (version 29.0.1) and aligned for figure configuration including labels and arrows. The line function tool was used to generate larger scale bars superimposed on the original scale bar embedded in the image from the QuPath output PNG for increased visibility.

References

1. Yushkevich PA, Piven J, Hazlett HC, et al. User-guided 3D active contour segmentation of anatomical structures: significantly improved efficiency and reliability. Neuroimage 2006;31:1116-1128.
2. Bankhead P, Loughrey MB, Fernandez JA, et al. QuPath: Open source software for digital pathology image analysis. Sci Rep 2017;7:16878.

Supplementary Table 1. ***Ex vivo*** **MRI protocol parameters for 3D gradient-recalled echo.**

Patient	1 naPPA/ CBS	2 naPPA/ CBS	3 naPPA/ PSP	4 naPPA/ PSP	5 svPPA/ bvFTD	6 Control
Isotropic Resolution (µm)	280	160	160	160	160	160
# of Gradient Echoes	8	3	3	3	3	3
Min. TE (ms)	3.48	9.42	9.42	9.37	9.42	9.42
Echo Spacing (ms)	6.62	11.34	11.34	11.34	11.34	11.34
TR (ms)	60	60	60	80	60	60
Flip Angle (deg)	25	25	25	25	25	25
Bandwidth (Hz/px)	400	90	90	90	90	90

Supplementary Table 2. Protocol parameters for T_1w and T_2w 3T and T_2*w 7T *in vivo* MRI images.

Patient	1 naPPA/ CBS	2 naPPA/ CBS	3 naPPA/ PSP	4 naPPA/ PSP	5 svPPA/ bvFTD	6 Control
3 TT_1w MPRAGE						
**FOV (mm)**	256 x 256 x 179	192 x 256 x 160	256 x 256 x 179	256 x 256 x 179		
**Resolution (mm)**	0.8 (iso)	1 (iso)	0.8 (iso)	0.8 (iso)		
**TI (ms)**	1020	950	1020	1020		
**TR (ms)**	2400	1620	2400	2400		
**Flip Angle (deg)**	8	15	8	8		
**Bandwidth (Hz/px)**	650 (4 echos)	150	650 (4 echos)	650 (4 echos)		
3T T_2w FLAIR TSE						
**FOV (mm)**	220 x 220 x 153		220 x 220 x 153	220 x 220 x 153		
**Resolution (mm)**	0.86 x 0.86 x 3		0.86 x 0.86 x 3	0.86 x 0.86 x 3		
**TI (ms)**	2500		2500	2500		
**TE (ms)**	88		88	88		
**TR (ms)**	9000		9000	9000		
**Flip Angle (deg)**	150		150	150		
**Echo Train Length**	19		19	19		
**Bandwidth (Hz/px)**	220		220	220		
3T T_2w TSE						
**FOV (mm)**		196 x 220 x 95				
**Resolution (mm)**		0.43 x 0.43 x 5				
**TE (ms)**		95				
**TR (ms)**		4000				

Supplementary Table 3. Diagnostic tau pathology severity scores.

Patient	1 naPPA/ CBS	2 naPPA/ CBS	3 naPPA/ PSP	4 naPPA/ PSP	5 svPPA/ bvFTD	6 Control
**Amygdala**	Rare	1+	2+	3+	3+	Rare
**Dentate Gyrus**	Rare	Rare	Rare	3+	3+	0
**CA-Subiculum**	1+	1+	3+	3+	3+	0
**Entorhinal**	1+	3+	1+	2+	3+	Rare
**Midfrontal**	Rare	3+	2+	3+	3+	0
**Angular**	Rare	2+	1+	3+	3+	0
**Superior/ Mid-temporal**	Rare	1+	Rare	2+	3+	0
**Anterior Cingulate**	1+	3+	2+	3+	3+	0
**Occipital**	0	Rare	Rare	Rare	1+	0
**Caudate/ Putamen**	2+	3+	2+	3+	3+	Rare
**Globus Pallidus**	1+	Rare	2+	2+	2+	0
**Thalamus**	Rare	2+	2+	3+	2+	0
**Midbrain**	2+	3+	2+	3+	3+	0
**Substantia Nigra**	2+	2+	3+	2+	3+	0
**Pons**	2+	2+	2+	2+	2+	0
**Locus Coeruleus**	2+	2+	NA	NA	2+	Rare
**Medulla**	2+	1+	2+	1+	2+	0
**Cerebellum**	Rare	1+	1+	1+	1+	0

0= none, rare= scant pathology, 1+=mild, 2+=moderate, 3+= severe. NA= not available

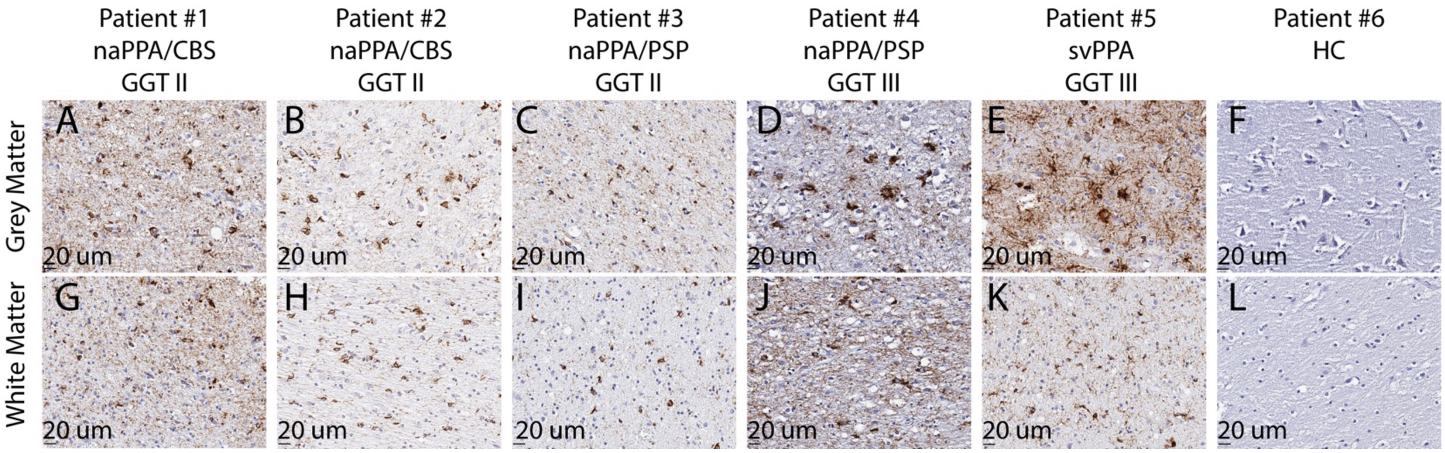

Supplementary Figure 1. Globular Glial Tauopathy (GGT) subtypes. Representative photomicrographs depicting AT8 tau immunostaining for morphology and ordinal ratings of relative globular oligodendrocytes and globular astrocytes in grey matter (top, A-F) and white matter (bottom, G-L). Primary motor cortex in Patients #1-3 with GGT subtype II (A-C and G-I), there were greater globular oligodendrocytes than globular astrocytes, while in Patients #4-5 (primary motor cortex in patient #4, D and J, anterior inferior temporal lobe in patient #5, E and K) with GGT subtype III there were greater globular astrocytes than globular oligodendrocytes. Patient #6 healthy control (HC) with absence of tau pathology in primary motor cortex F and L. Scale bar=20 µm. naPPA= nonfluent variant of primary progressive aphasia, CBS=corticobasal syndrome, PSP= progressive supranuclear palsy, svPPA= semantic variant of primary progressive aphasia, HC= healthy control.

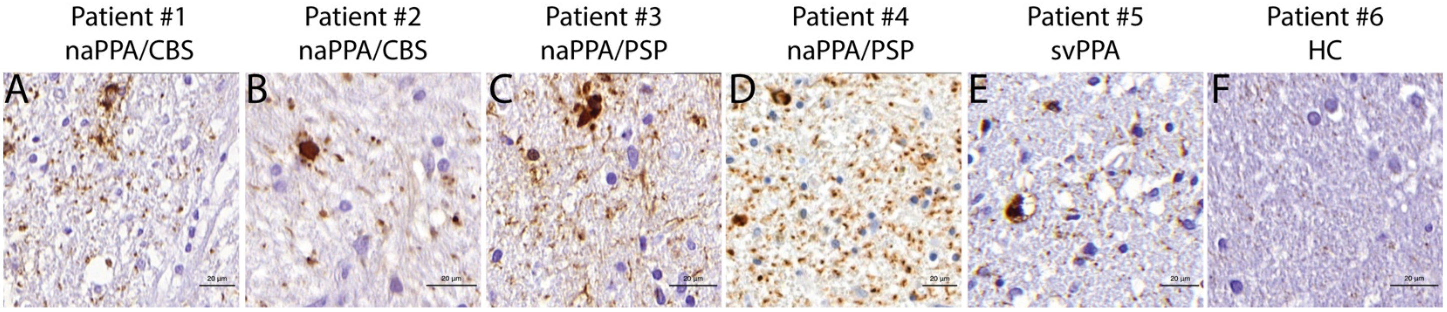

Supplementary Figure 2. Corticospinal tract involvement in Globular Glial Tauopathy (GGT). Representative photomicrographs depicting tau immunostaining (PHF-1 antibody) from diagnostic slides for ordinal ratings of tau pathology in the corticospinal tract in the cerebral peduncle at the level of the superior colliculus of the midbrain. A= Patient #1 with non-fluent variant of primary progressive aphasia (naPPA) and corticobasal syndrome (CBS) clinical features, B= Patient #2 with naPPA/CBS, C=Patient #3 with naPPA and progressive supranuclear palsy (PSP) syndrome, D=Patient #4 with naPPA/PSP, E=Patient #5 with semantic variant primary progressive aphasia (svPPA), F=Patient #6 healthy control (HC). Scale bar=20 µm.

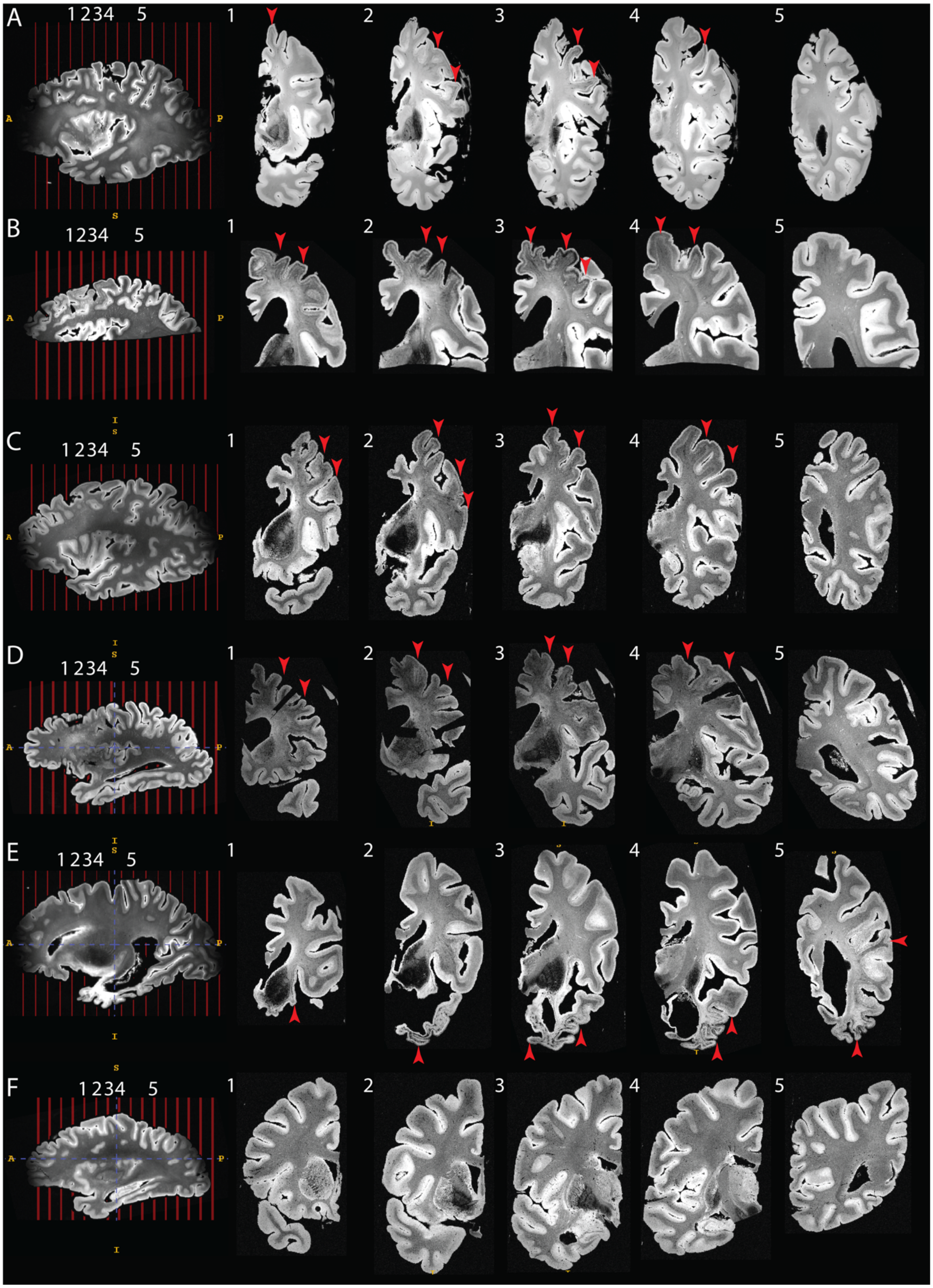

Supplementary Figure 3. Whole Hemisphere Ex vivo 7T T_2*w MRI. Sagittal views of whole-hemisphere ex vivo 7T T_2*w MRI and corresponding coronal views at slices 1-5 depicting cortical distribution of hypointense cortical signal (arrows) for non-fluent primary progressive aphasia (naPPA) patients #1-4 (panels A-D), semantic variant of primary progressive aphasia (svPPA) patient #5 (panel E) which are absent in the control patient #6 (panel F). Samples are oriented in radiological convention for left/right

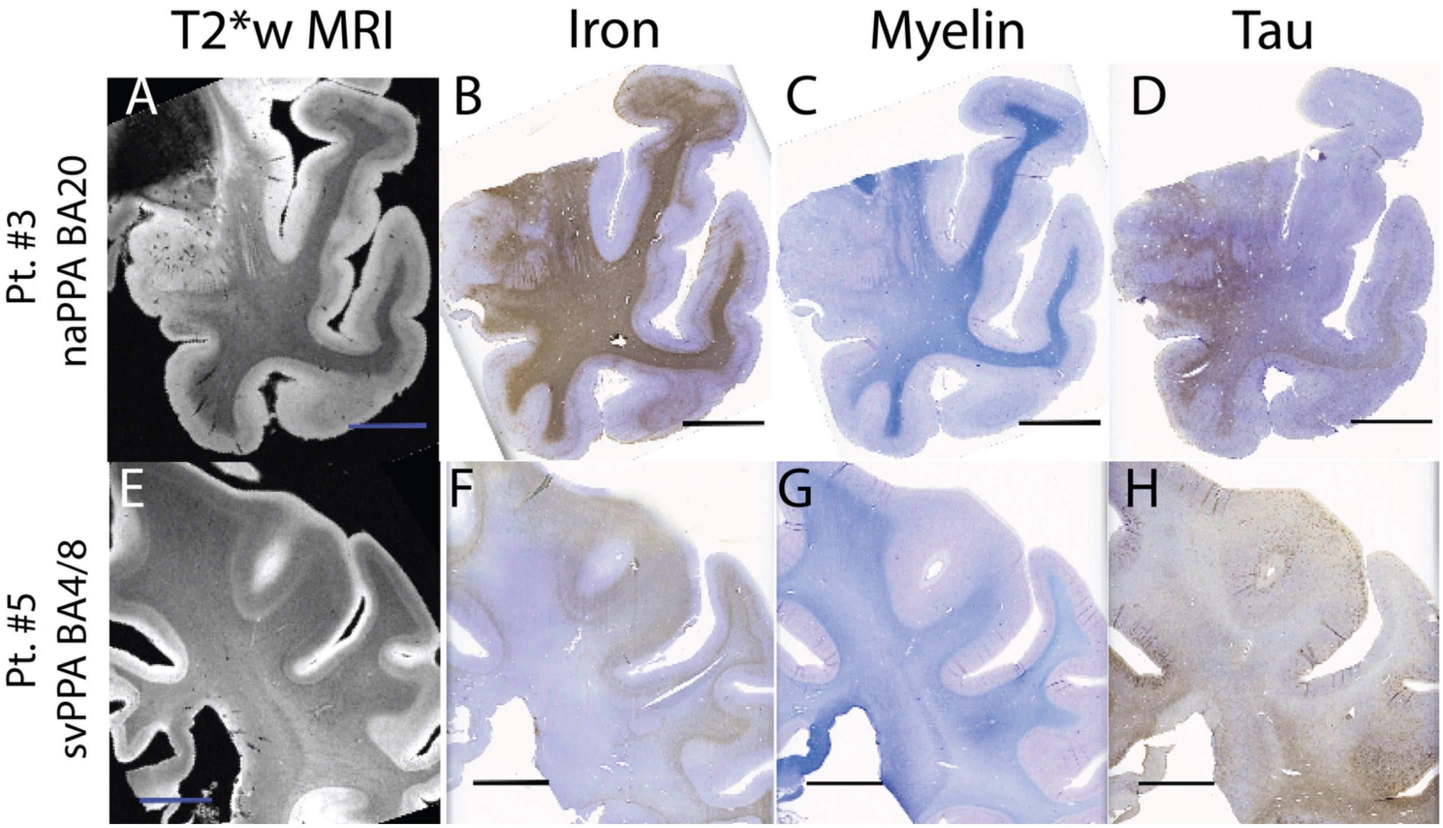

Supplementary Figure 4. Histopathological analysis and paired ex vivo 7T T_2*w MRI in low pathology regions. Examples of ex vivo MRI (7T2*w) and corresponding histopathology in low disease pathology regions in (A-D) non-fluent primary progressive aphasia (naPPA) patient #3 (BA20) and (E-H) in semantic variant of primary progressive aphasia (svPPA) patient #5 (BA4/8) show mild tau pathology with AT8 immunostain (D, H), with preserved cortical myelin and neuronal columnar organization with luxol-fast blue/cresyl-violet staining (C,G) lacking significant iron-rich gliosis on DAB-enhanced Perl's stain (B,F). Scale bar= 1 cm.

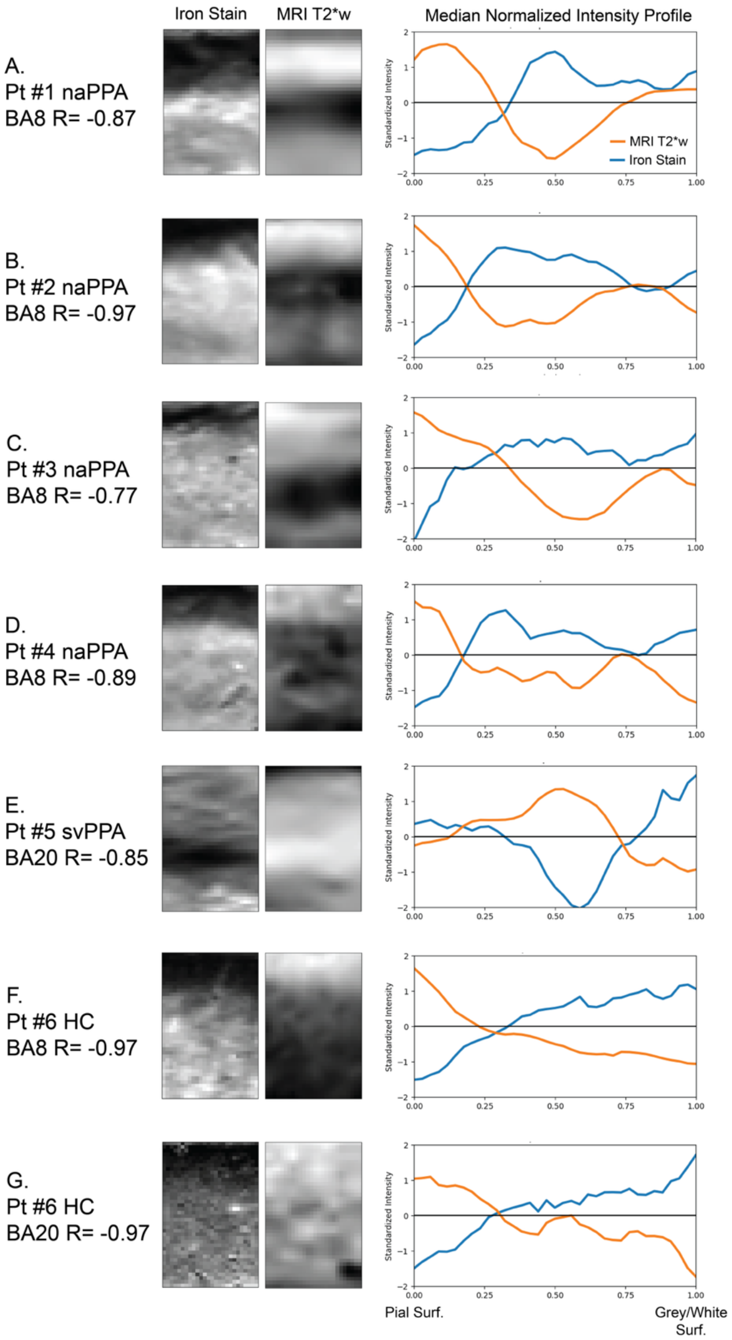

Supplementary Figure 5. Laminar intensity profiles of 7T2*w MRI and corresponding DAB-enhanced iron stain in tissue. Regions of interest were manually annotated in representative cortex for both DAB-enhanced iron stain tissue and corresponding 7T2*w MRI in BA 8 for naPPA patients #1-4 (A-D) and control patient #6 (F) and BA20 for svPPA patient #5 (E) and control patient #6 (G) for extraction of grey-scale intensity of both 7T2*w MRI and corresponding iron-stained tissue. Intensity is plotted in normalized distance from pial surface to grey-white boundary. Standardized intensity values on the y-axis plotted along the cortical depth on the x-axis from pial surf. (surface) (left) to grey-white surf. boundary (right) for 7T2*w MRI (orange) and corresponding DAB-enhanced iron-stain (blue). Pearson R for intensity values from 100 points in normalized space was calculated for each image-pair finding strong inverse correlation (i.e. decreasing hypointense signal on 7T2*wMRI with increasing DAB intensity on tissue; average Pearson R=-0.90+0.08, n=7 image-pairs, p<0.001) confirming observations of mid-to-deep cortical layer iron-rich gliosis association with pathological mid-to-deep cortical layer irregular hypointense band on ex vivo 7T2*w MRI in GGT patients. Draw-redraw validation finds similar mean R=-0.78+0.16, n=7 image-pairs, p<0.001; data not shown- see data availability statement. naPPA= nonfluent variant of primary progressive aphasia, svPPA= semantic variant of primary progressive aphasia, HC= healthy control.

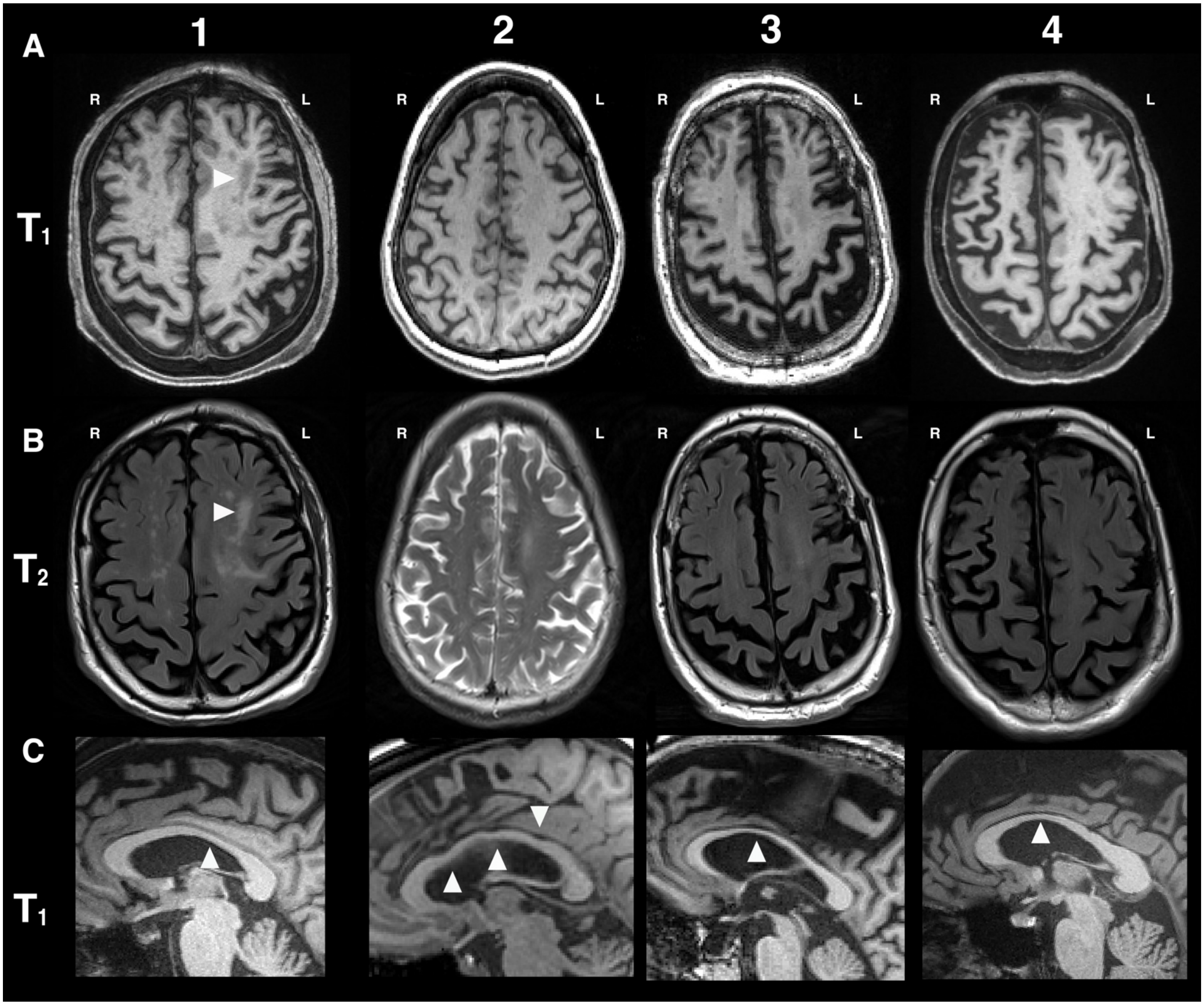

Supplementary Figure 6. Antemortem 3T T_1w and T_2w MRI for nonfluent primary progressive aphasia (naPPA) patients #1-4. NaPPA patients #1-4 (columns) axial views through superior aspect of brain, aligned to the native acquisition plane of the T_2w scan. T_1w (A) and T_2w MRI (B) find variable frontal cortical atrophy and prominent white matter hyperintensity on T_2W MRI in patient #1 (B, arrow). Sagittal T_1w MRI views (C) find thinning in mid-to-posterior aspects of the corpus callosum (arrows).
